# Thalamic nuclei segmentation from T1-weighted MRI: Unifying and benchmarking state-of-the-art methods

**DOI:** 10.1162/imag_a_00166

**Published:** 2024-05-08

**Authors:** Brendan Williams, Dan Nguyen, Julie P. Vidal, Manojkumar Saranathan

**Affiliations:** Centre for Integrative Neuroscience and Neurodynamics, University of Reading, Reading, United Kingdom; School of Psychology and Clinical Language Sciences, University of Reading, Reading, United Kingdom; Department of Radiology, University of Massachusetts Chan Medical School, Worcester, MA, United States; CNRS, CerCo (Centre de Recherche Cerveau et Cognition) - Université Paul Sabatier, Toulouse, France; INSERM, ToNiC (Toulouse NeuroImaging Center) - Université Paul Sabatier, Toulouse, France

**Keywords:** thalamus, segmentation, FreeSurfer, THOMAS, human connectome project, Alzheimer’s disease

## Abstract

The thalamus and its constituent nuclei are critical for a broad range of cognitive, linguistic, and sensorimotor processes, and are implicated in many neurological and neurodegenerative conditions. However, the functional involvement and specificity of thalamic nuclei in human neuroimaging work is underappreciated and not well studied due, in part, to technical challenges of accurately identifying and segmenting nuclei. This challenge is further exacerbated by a lack of common nomenclature for comparing segmentation methods. Here, we use data from healthy young (Human Connectome Project, n = 100) and older healthy adults, plus those with mild cognitive impairment and Alzheimer’s disease (Alzheimer’s Disease Neuroimaging Initiative, n = 540), to benchmark four state-of-the-art thalamic segmentation methods for T1 MRI (FreeSurfer, histogram-based polynomial synthesis [HIPS]-THOMAS, synthesized contrast segmentation [SCS]-convolutional neural network [CNN], and T1-THOMAS) under a single segmentation framework. Segmentations were compared using overlap and dissimilarity metrics to the Morel stereotaxic atlas, a widely accepted thalamic atlas. We also quantified each method’s estimation of thalamic nuclear degeneration across Alzheimer’s disease progression, and how accurately early and late mild cognitive impairment, and Alzheimer’s disease could be distinguished from healthy controls. We show that the HIPS-THOMAS approach produced the most effective segmentations of individual thalamic nuclei relative to the Morel atlas, and was also most accurate in discriminating healthy controls from those with mild cognitive impairment and Alzheimer’s disease using individual nucleus volumes. This latter result was different when using whole thalamus volumes, where the SCS-CNN approach was the most accurate in classifying healthy controls. This work is the first to systematically compare the efficacy of anatomical thalamic segmentation approaches under a unified nomenclature. We also provide recommendations of which segmentation method to use for studying the functional relevance of specific thalamic nuclei, based on their overlap and dissimilarity with the Morel atlas.

## 1 Introduction

Accurate identification of thalamic nuclei across spatial scales is important due to their widespread involvement in an array of functions, including sensory perception, motor control, sleep and arousal, and linguistic, memory, and cognitive processes (Boeken et al., 2023;Chakraborty et al., 2016;Greene et al., 2020;Setzer et al., 2022;Williams & Christakou, 2021;Wolff & Vann, 2019;Yang et al., 2020). Aberrant thalamic structure and function are also implicated in a broad range of neurological, neuropsychiatric, developmental, and neurodegenerative conditions, including epilepsy, schizophrenia, autism spectrum disorder, multiple sclerosis, and Alzheimer’s disease (Aggleton et al., 2016;DeNicola et al., 2020;Elvsåshagen et al., 2021;Fu et al., 2019;Giraldo-Chica et al., 2018;Liebermann et al., 2013;Maximo & Kana, 2019;Papathanasiou et al., 2015;Shao et al., 2013;Smith et al., 2014;Whiting et al., 2018). However, most MRI-based analyses treat the thalamus as a homogenous entity, reducing sensitivity to thalamic nuclei-specific effects. Furthermore, thalamic nuclei segmentation from anatomical T1- and T2-weighted MRI data has been hampered by suboptimal image contrast, resulting in poor delineation of intrathalamic and whole thalamus boundaries. Instead, most thalamic nuclei segmentation methods, to date, have been based on Diffusion Tensor Imaging (DTI), which is limited by the lack of anisotropy in the largely grey-matter dominant thalamus, and functional MRI, which is limited by poor spatial resolution and distortion of the underlying echoplanar imaging acquisition (Behrens et al., 2003;Johansen-Berg et al., 2005;Kim et al., 2013;Kumar et al., 2017;Wiegell et al., 2003;Zhang et al., 2008). As a result, these methods do not resolve small structures such as lateral and medial geniculate nuclei (LGN/MGN), and the anteroventral (AV) nucleus, which are critical for sensory and cognitive processing.

Due to its inclusion in most publicly available datasets and neuroimaging protocols, alongside its high isotropic spatial resolution (usually 1 mm or better), there has been a renewed interest in thalamic segmentation based on anatomical T1-weighted (T1w) MRI, despite its poor contrast in the thalamus. Recently introduced thalamic segmentation methods like the FreeSurfer Bayesian inference (Iglesias et al., 2018), and the THOMAS multi-atlas (Su et al., 2019) approaches have been used to analyze data in disease states like Alzheimer’s disease, alcohol use disorder, and multiple sclerosis (Abuaf et al., 2022;Bonham et al., 2023;Forno et al., 2023;Zahr et al., 2020). While FreeSurfer primarily works on T1 Magnetization Prepared RApid Gradient Echo (MPRAGE) data with the ability to incorporate secondary images with different image contrast (Tregidgo et al., 2023), the original THOMAS algorithm (Su et al., 2019) was optimized and validated using white-matter-nulled (WMn) MPRAGE, a special pulse sequence that nulls white-matter instead of cerebrospinal fluid (CSF) as in standard MPRAGE. THOMAS was recently adapted for conventional T1w MRI in a modified method (T1-THOMAS), using a mutual information (MI) metric for nonlinear registration and a majority voting (MV) algorithm for label fusion. However, T1-THOMAS was not as accurate compared to segmentations based on WMn-MPRAGE for several small nuclei, presumably due to loss of intrathalamic contrast and poor delineation of thalamic boundaries in T1w MPRAGE contrast (Bernstein et al., 2021). To leverage the improved intrathalamic contrast of WMn imaging, a deep learning-based approach has been proposed using a first convolutional neural network (CNN) to synthesize WMn-MPRAGE-like images from T1w-MRI and a second CNN to perform segmentation on the synthesized WMn images. This method, called synthesized contrast segmentation (SCS), was shown to be much more accurate than direct CNN segmentation of T1w-MRI data (Umapathy et al., 2022). Another recent method uses a robust histogram-based polynomial synthesis (HIPS) approach instead of a CNN for the synthesis of WMn-MPRAGE-like images, and those synthesized images are then identically processed as in the original THOMAS method for thalamic nuclei segmentation (Vidal et al., 2024). This method also showed significant improvement in Dice and reduction in volume errors compared to T1-THOMAS and was demonstrated to be more robust than the SCS-CNN when applied to data from higher field strengths or scanner manufacturers that were not part of the CNN training process (Vidal et al., 2024).

A widely used reference guide for identifying thalamic nuclei is the Morel stereotaxic atlas (Morel, 2007;Morel et al., 1997), which was developed using histological staining of five post-mortem brains from healthy older adults for the calcium binding proteins parvalbumin, calbindin D-28k, and calretinin to identify cyto- and myeloarchitectural features. Functional relevance was also considered during the development of the Morel atlas. More recently, the Morel atlas has been digitized (Krauth-Morel atlas) and made available in MNI-space for use as a potential reference in a wide variety of neuroimaging applications (Krauth et al., 2010). However, despite their claims of conformity with the Morel atlas, both FreeSurfer and THOMAS use slightly different nomenclatures and definitions for thalamic nuclei (Freesurfer followsJones (2012)nomenclature while THOMAS follows Morel nomenclature) and produce parcellations which, at first glance, differ qualitatively from each other. As a result, direct comparisons of these segmentation methods on the same datasets have not been reported. Here, we systematically compare four state-of-the-art methods for thalamic nuclei segmentation of T1w MRI: FreeSurfer, HIPS-THOMAS, SCS-CNN, and T1-THOMAS. We used data from healthy younger adults in the Human Connectome Project (HCP) to quantitatively compare segmentations from these methods against the Krauth-Morel atlas (Krauth et al., 2010) in subject-space as well as MNI-space. We then analyzed data from older adults from the Alzheimer’s Disease Neuroimaging Initiative (ADNI) database to characterize thalamic atrophy as a function of disease status and assessed the accuracy of each of the methods in predicting Alzheimer’s disease status using a receiver operating characteristic (ROC) analysis.

## 2 Methods

### 2.1 Participants

Anatomical T1w-MRI data were sourced from two publicly available neuroimaging datasets: the Human Connectome Project (HCP) (Van Essen et al., 2013) and Alzheimer’s Disease Neuroimaging Initiative (ADNI) (http://adni.loni.usc.edu). 100 subjects were pseudo-randomly selected from the HCP dataset as in previous work (Williams et al., 2022). A subset of 540 subjects were selected from the ADNI dataset, comprising participants who had undergone a Montreal Cognitive Assessment (MoCA) test and were scanned on 3T MRI using MPRAGE followed by successful image registration and segmentation (seeBernstein et al., 2021for further details). Subjects were classified as either healthy control (HC, 119 subjects), early mild cognitive impairment (EMCI, 208 subjects), late mild cognitive impairment (LMCI, 116 subjects), or Alzheimer’s disease (AD, 97 subjects). EMCI and LMCI were classified based on subjective memory concern scores (either themselves, their partner, or a clinician) from the logical memory II subscale of the Wechsler Memory Scale—Revised (16+ years in education: EMCI scores 9–11, LMCI > 4 & ≤ 8; 8–15 years in education: EMCI scores 5–9, LMCI > 2 & ≤4; 0–7 years in education: EMCI scores 3–6, LMCI > 0 & ≤2), a Mini-Mental State Examination Score between 24–30, a Clinical Dementia Rating of 0.5 in the memory box, and sufficient cognitive and functional performance that would not make threshold for an AD diagnosis (Bernstein et al., 2021).

### 2.2 Data processing

#### 2.2.1 Pre-processing

HCP data (n = 100) were pre-processed using the HCP minimal preprocessing pipelines (Glasser et al., 2013). Firstly, T1w images were corrected for gradient distortions using a customized version of*gradient_nonlin_unwarp*in FreeSurfer, then each subject’s two T1w scans were aligned using FSL FLIRT and averaged. The averaged T1w image was then registered to MNI-space using a 12 DOF affine registration with FLIRT, and a subset of 6 DOF transforms were used to align the anterior commissure, the anterior commissure–posterior commissure line, and the inter-hemispheric plane, while preserving the size and shape of the brain in native space. The skull was removed by inverting linear (FLIRT) and nonlinear (FNIRT) warps from anatomical to MNI-space, applying the warp to the MNI-space brain mask, and then applying the mask to the averaged T1w image. Finally, the image was corrected for readout distortion and biases in B_1_and B_1_^+^fields. T1w MPRAGE datasets from ADNI (n = 540) were directly processed using the different thalamic nuclei segmentation methods with no extra pre-processing steps. Note that the N4 bias correction to remove shading is incorporated inside the THOMAS and SCS-CNN pipelines.

#### 2.2.2 Thalamic nuclei segmentation

The four main thalamic segmentation schemes compared in this work are summarized inFigure 1and described below.

**Fig. 1.**
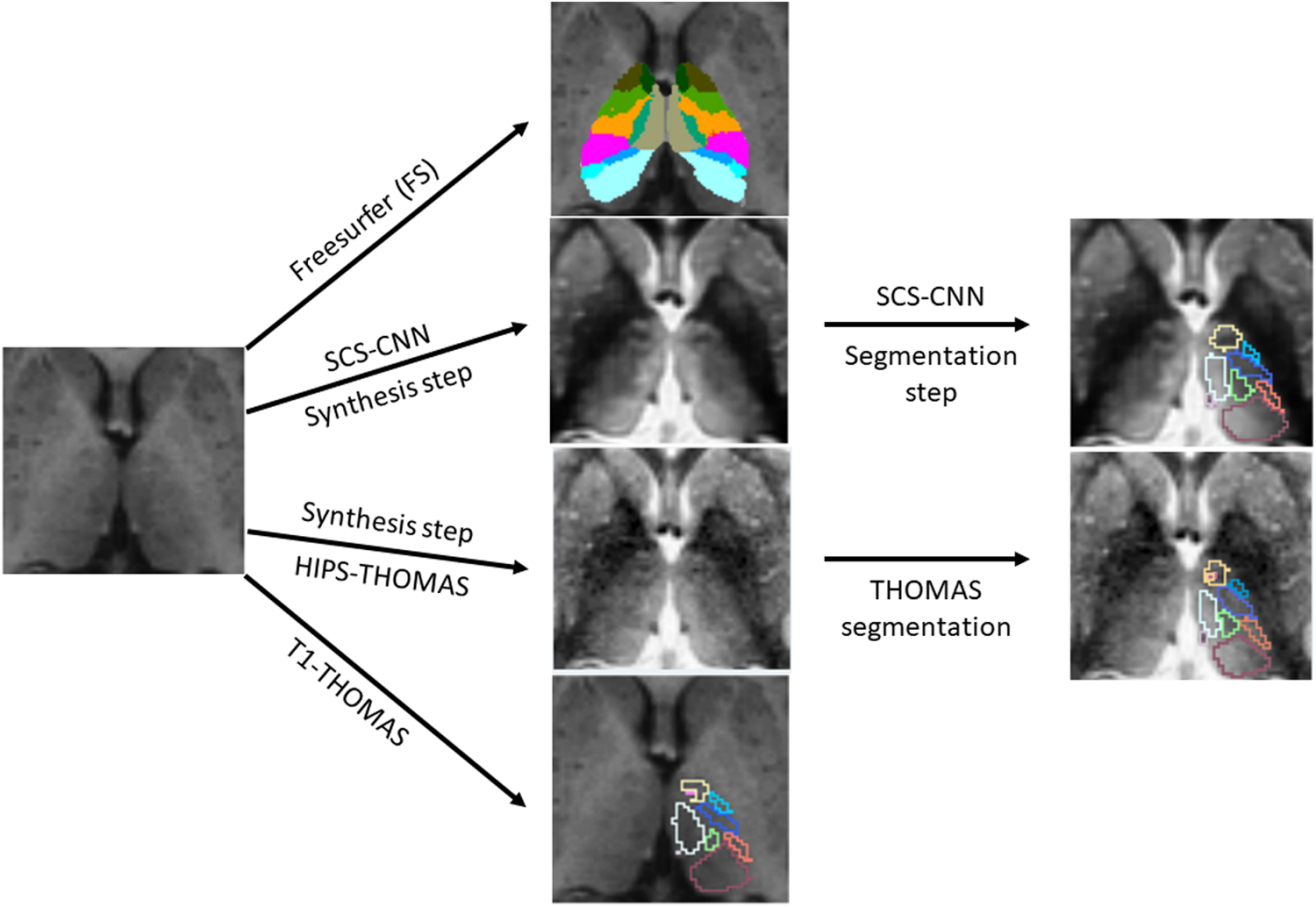
Overview of thalamic nuclei segmentation used for HCP and ADNI T1w-MRI datasets showing the FreeSurfer, HIPS-THOMAS, SCS-CNN, and T1-THOMAS schemes.

##### 2.2.2.1 Freesurfer

HCP and ADNI data were segmented following methods described previously (Iglesias et al., 2018;Williams et al., 2022). Data processing was run using a Nipype pipeline integrating FSL (version 6.0.4) and FreeSurfer (version 7.1.1). Anatomical T1w images were first processed and parcellated using*recon-all*in FreeSurfer; the output of recon-all was used to initialize the parcellation of thalamic nuclei for anatomical data using the algorithm described byIglesias et al. (2018). The parcellated thalamus was converted from FreeSurfer space to native anatomical space and changed from mgz to nii file format using*mri_label2vol*and*mri_convert*in FreeSurfer, respectively.

##### 2.2.2.2 THOMAS variants

T1w MRI datasets from both the HCP and ADNI databases were segmented using three THOMAS variants. The first, T1-THOMAS, is an adaptation of the original THOMAS method for T1w-MRI that uses a mutual information metric for nonlinear registration and majority voting for label fusion (Bernstein et al., 2021). The second, a synthesized contrast segmentation convolutional neural network (SCS-CNN), uses two CNNs to generate segmentations (Umapathy et al., 2022). The first CNN was trained using patches from contemporaneously acquired T1w and WMn-MPRAGE data and is used to synthesize WMn-like images from T1w data. The second CNN was trained using WMn-MPRAGE data labeled using THOMAS and is used to segment the synthesized WMn-like image generated by the first CNN. The third, HIPS-THOMAS, incorporates a histogram-based polynomial synthesis preprocessing step using information from the histograms and a plot of each voxel’s intensities of T1w and WMn-MPRAGE images to compute a polynomial approximation from a small subset of training images used to synthesize WMn-like images from T1w (Vidal et al., 2024). More information and schematics can be found in theSupplementary MethodsandSupplementary Figure 1. Importantly, the original THOMAS algorithm was developed using manual segmentations of WMn-MPRAGE data guided using the Morel atlas (Su et al., 2019).

### 2.3 Label synthesis and segmentation preparation

For FreeSurfer outputs and the Krauth-Morel atlas, thalamic nuclei were combined to match the Morel nomenclature used by THOMAS to generate 10 thalamic nuclei for subjects in both the HCP and ADNI datasets (Table 1). Note that habenula and mammillothalamic tract (MTT) were omitted as FreeSurfer did not segment those structures. Similarly, lateral nuclei such as lateral dorsal and intralaminar nuclei such as centrolateral which are not segmented by THOMAS were omitted. For the HCP subjects, rigid and affine transformations, and non-linear warps between native subject space and MNI-space were generated with the Advanced Normalization Tools (ANTs) package (Version 2.3.5, Ecphorella) (Avants et al., 2008). These image transformations and warps were used to generate subject space versions of Krauth-Morel nuclei, and MNI-space versions of FreeSurfer, SCS-CNN, and HIPS-THOMAS segmented nuclei for comparison using nearest neighbour interpolation in ANTs. The MNI-space versions of the segmented nuclei were used to generate group-level probabilistic atlases (Najdenovska et al., 2018).

**Table 1.** Method for combining FreeSurfer and Krauth-Morel nuclei to match the Morel nomenclature used by THOMAS, producing a unified space for thalamic segmentation comparison.

THOMAS nuclei	FreeSurfer nuclei	Krauth-Morel nuclei
AV	AV	AV
VA	VAmc + VApc	VAmc + VApc
Vla	Vla	Vla
VLp	VLp	VLpd + VLpv + VLp
VPL	VPL	VPLa + VPLp
Pul	PuA + PuI + PuL+ PuM	PuA+ PuI+ PuL+ PuM
LGN	LGN	LGNmc + LGNpc
MGN	MGN	MGN
CM	CM	CM
MD-Pf	MDl+ MDm + Pf	Pf + sPf + MDmc + MDpc

### 2.4 Segmentation metrics

Dice similarity coefficients (Dice) were used to compare the 10 segmented thalamic nuclei per hemisphere from each approach with the Krauth-Morel atlas in both subject space and MNI-space for the HCP data. Dice is a widely used measure in image processing for assessing overlap, and is defined as:

$$Dice\left(S_{x},S_{y}\right)=\frac{2\left|S_{x}\cap S_{y}\right|}{\left|S_{x}\right|+\left|S_{y}\right|}$$

where$\left|S_{x}\cap S_{y}\right|$is the cardinality of the intersection between the segmentation and ground truth (this is equal to the number of true positives, or overlapping voxels), divided by the sum of the cardinality of the ground truth$\left|S_{x}\right|$and the segmentation$\left|S_{y}\right|$(equal to the sum of true positives, false positives, and false negatives) (Williams et al., 2022). Using subject space data, we ranked each segmentation approach from best to worst (1 to 4) for each of the nuclei using paired-sample t-test results. For the MNI-space analysis, a threshold of 0.25 was used to binarize the group-level probabilistic atlas. We used the following cut-offs to compare segmentations: Dice = 0 no agreement, 0 < Dice < 0.2 slight agreement, 0.2 ≤ Dice < 0.4 fair agreement, 0.4 ≤ Dice < 0.6 moderate agreement, 0.6 ≤ Dice < 0.8 substantial agreement, and 0.8 ≤ Dice ≤ 1 almost perfect agreement (Pajula et al., 2012).

We also calculated the Average Hausdorff Distance (AHD) to compare the 10 segmented thalamic nuclei with the Krauth-Morel atlas. The Average Hausdorff Distance is used as a measure of dissimilarity and can account for differences in isometry. Distance-based metrics are advantageous relative to overlap-based metrics in situations where segmentations are small because overlap-based metrics disproportionately penalize errors for smaller versus larger segmentations, as is the case with thalamic nuclei segmentations (Taha & Hanbury, 2015). In relation to image segmentation, the Hausdorff Distance can be defined as the minimum number of voxels between a point in segmentation X and a point in segmentation Y. Therefore, the Average Hausdorff Distance is the average minimum distance between all points in segmentation A and segmentation B in voxels. The Average Hausdorff Distance is defined as:

Average Hausdorff Distance(A,B)=max(d(A,B),d(B,A))

$$d(A,B)=\frac{1}{N}\sum_{a\in A}\underset{b\in B}{\min}||a-b||$$

whered(A,B)is the average minimum distance (min ‖a−b‖) from voxels in the ground truth (A) to the segmentation (B),d(B,A)is the average minimum distance (min ‖a−b‖) from voxels in the segmentation (B) to the ground truth (A). The Average Hausdorff Distance is then the maximum of either of these two average distance measures in voxels. Although—to the best of our knowledge—no consensus guidelines exist for AHD thresholds, any segmentation with a coefficient < 1 voxel distance could be considered as a “good” segmentation since segmented voxels, on average, have at least partial overlap with voxels in the ground truth.

#### 2.4.1 Identifying nucleus-wise “best” segmentation methods

We initially defined the best segmentation approach for each nucleus based on subject-space and MNI-space results. A single segmentation was defined as the best for a given nuclei if it had the highest MNI-space Dice coefficient and had a significantly higher Dice coefficient in the subject-space analysis than other approaches. Two segmentations were defined jointly as being the best if they (a) had non-significant differences at the subject level and different directions of Dice coefficients results for subject- and MNI-space data, or (b) had significantly different scores at the subject level but the direction for the group Dice coefficient showed the inverse effect to the result of the null hypothesis significance test. If more than two segmentations met the previous criteria, then no segmentation approach was defined as the best overall. We then corroborated Dice coefficient results with AHD measures.

### 2.5 Statistical analysis

We performed statistical analyses using R and jamovi. We used two-way ANOVAs to compare segmentation metrics (Dice coefficient and Average Hausdorff Distance) between different segmentation methods in subject space, testing for main effects of segmentation methods (FreeSurfer, HIPS-THOMAS, SCS-CNN, and T1-THOMAS), hemisphere (left and right), and for interactions between segmentation methods and hemispheres. Post hoc t-tests (Bonferroni corrected) were used to compare between segmentation methods for each nucleus. We used ANCOVAs to compare nuclei volumes for each segmentation method using the ADNI dataset to test for main effects of group (HC, EMCI, LMCI, AD), and included age and intracranial volume (eTIV output of FreeSurfer) as covariates. Dunnett’s test was used for post hoc analyses to compare HC with EMCI, LMCI, and AD groups. We obtained least-squares estimates for volumes after adjusting for covariates, and effects with adjusted p < 0.05 were considered statistically significant. To calculate effect sizes for each pairwise comparison, we computed Cohen’s d. Lastly, we performed a Receiver Operating Characteristic (ROC) analysis for the ADNI dataset using logistic regression to quantify the ability of each segmentation method (nuclei volumes) to discriminate EMCI, LMCI, and AD from HC. We calculated area under the curve (AUC) values for each of the three scenarios for the different segmentation methods. AUCs were also computed using whole thalamus volumes alone for comparison.

## 3 Results

### 3.1 Human connectome project

#### 3.1.1 Subject-space analysis

**Fig. 2.**
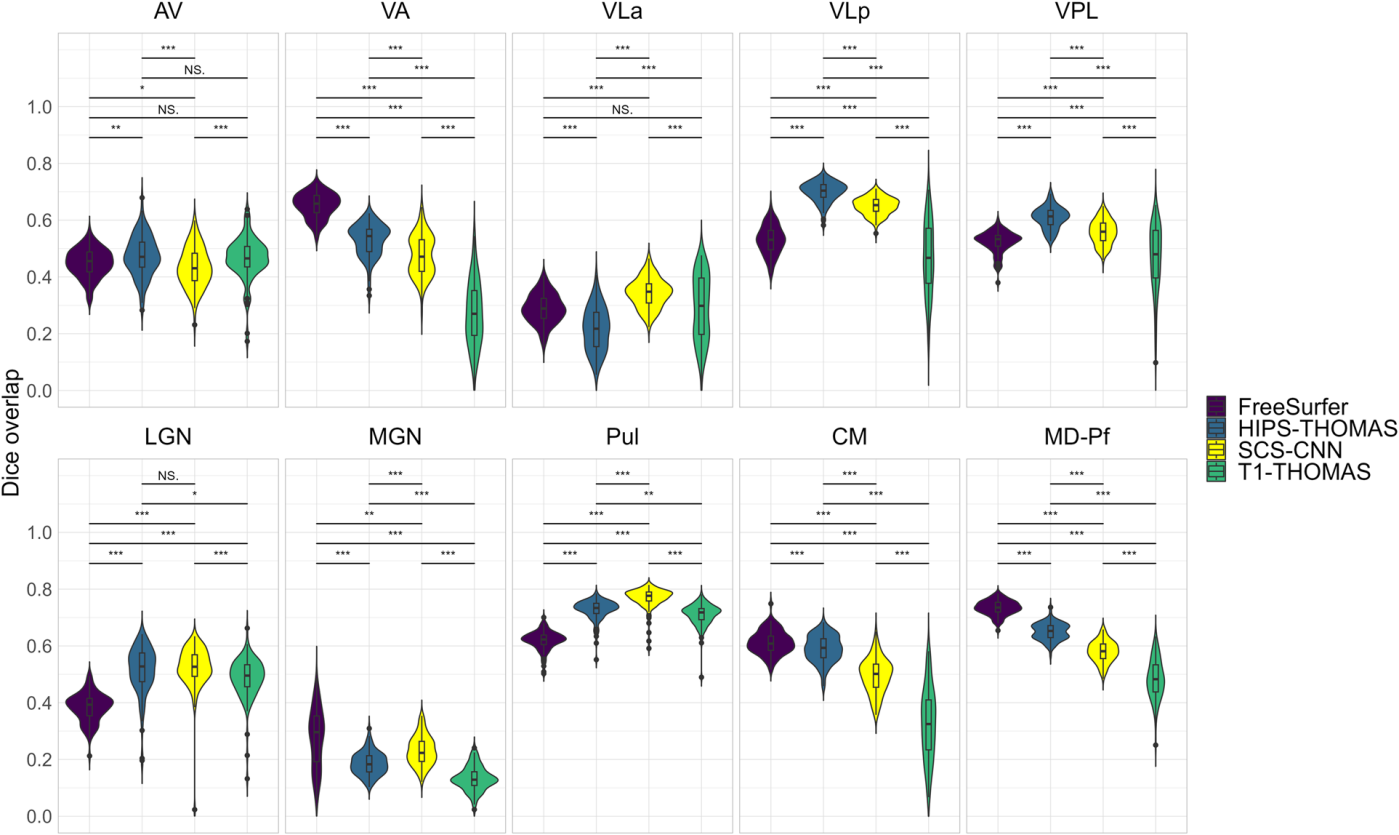
Violin plots of Dice overlap between left hemisphere nuclei segmented from Human Connectome Project data using FreeSurfer, HIPS-THOMAS, CNN-SCS, and T1-THOMAS approaches. Post hoc t-test results (Bonferroni corrected) are presented to show pairwise difference between segmentation approaches for each nucleus (*p < 0.05, **p < 0.01, and ***p < 0.001).

**Fig. 3.**
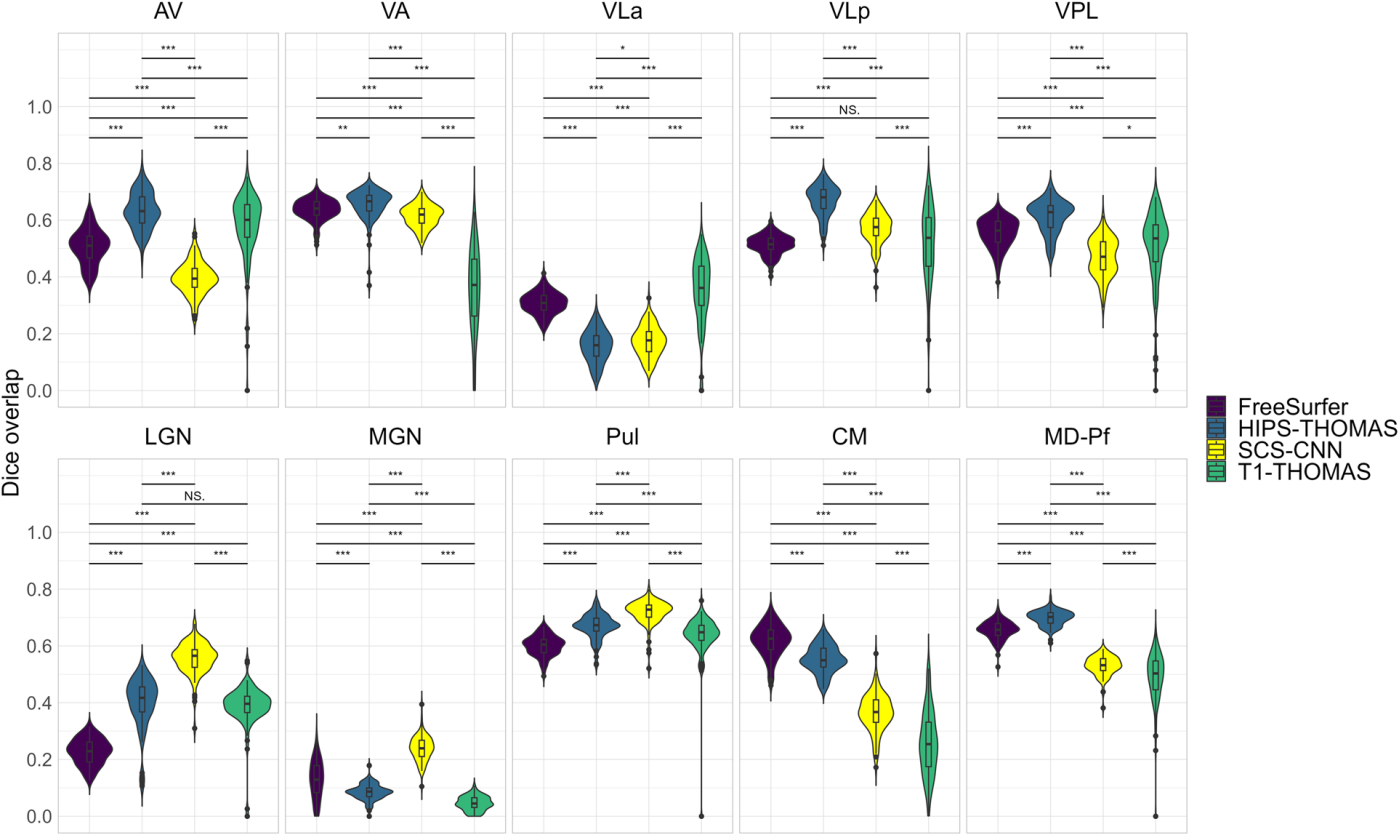
Violin plots of Dice overlap between right hemisphere nuclei segmented from Human Connectome Project data using FreeSurfer, HIPS-THOMAS, CNN-SCS, and T1-THOMAS approaches. Post hoc t-test results (Bonferroni corrected) are presented to show pairwise difference between segmentation approaches for each nucleus (*p < 0.05, **p < 0.01, and ***p < 0.001).

Two-way ANOVAs found significant main effects of segmentation approach and hemisphere for all nuclei (except for VPL, which had a non-significant main effect of hemisphere), and a significant interaction between the segmentation approach and hemisphere for all nuclei (Supplementary Table 1). Post hoc t-tests (Bonferroni corrected) for main effects of segmentation approach on Dice coefficients are summarized inFigure 2for left thalamic nuclei andFigure 3for right thalamic nuclei. To compare segmentation approaches based on the subject-space Dice coefficients, we ranked each segmentation approach from best (1) to worst (4) based on the post hoc t-tests for main effects of segmentation approach. Overall, HIPS-THOMAS had the best (lowest) mean ranking (L 1.9, R 1.8), followed by the FreeSurfer (L 2.1, R 2.5) and SCS-CNN approaches (L 2.2, R 2.5), and then THOMAS (L 3.3, R 3). HIPS-THOMAS also had the lowest overall variation in ranking (L SD = 0.899, R SD = 0.934), as shown by a smaller standard deviation compared to the other approaches (FreeSurfer L SD = 1.164, R SD = 0.879; SCS-CNN L SD = 0.934, R SD = 1.066; T1-THOMAS L SD = 0.958, R SD = 0.953). We calculated the Average Hausdorff Distance in voxels between the segmented nuclei and the corresponding nuclei from the Krauth-Morel atlas in subject-space for left and right nuclei. We also used two-way ANOVAs to compare Average Hausdorff Distances between segmentation approaches and hemisphere for each thalamic nucleus. We found a significant main effect of the segmentation approach for VA (*F*(1, 98.15) = 9.886, p = 0.002, η_g_^2^= 0.037), VLp (*F*(1, 98.26) = 4.052, p = 0.047, η_g_^2^= 0.015), LGN (*F*(1.01, 98.89) = 5.02, p = 0.027, η_g_^2^= 0.019), MGN (*F*(1.02, 99.95) = 10.816, p = 0.001, η_g_^2^= 0.04), CM (*F*(1, 98.32) = 6.062, p = 0.015, η_g_^2^= 0.023), and MD-Pf (*F*(1, 98.17) = 4.28, p = 0.041, η_g_^2^= 0.016), but no significant main effects of the segmentation approach for AV, VLa, VPL, or Pul. We found a significant main effect of hemisphere for LGN (*F*(1, 98) = 5.674, p = 0.019, η_g_^2^= 0.007) and MGN (*F*(1, 98) = 11.223, p = 0.001, η_g_^2^= 0.014), but not for other thalamic nuclei (Supplementary Figs. 2 & 3). We found no significant interactions between the segmentation approach and hemisphere. Post hoc t-tests (Bonferroni corrected) for main effects of the segmentation approach on Average Hausdorff Distances are summarized inSupplementary Figures 2 and 3for left and right thalamic nuclei, respectively. Like for Dice, we ranked each segmentation approach from best (lowest AHD) to worst (highest AHD). Using only nuclei where there was a main effect of the segmentation approach on AHD, HIPS-THOMAS had the best (lowest) mean ranking (L & R 1.67), followed by FreeSurfer in the left hemisphere (1.83) and SCS-CNN in the right hemisphere (2). Both FreeSurfer and SCS-CNN had rankings of 2.3 in the right and left hemispheres, respectively. Bilaterally, THOMAS had the worst mean ranking (L 3.83, R 3.3). The lowest variation in rankings was found for THOMAS in the left hemisphere (SD = 0.37); the next lowest variation (SD = 0.75) was found bilaterally for HIPS-THOMAS, and in the left and right hemispheres for SCS-CNN and THOMAS, respectively. Bilaterally, FreeSurfer had the greatest variation in rankings (L SD = 1.21, R SD = 0.94).

#### 3.1.2 MNI-space analysis

Dice and Hausdorff-distance metrics computed in MNI-space are tabulated for the left and right hemispheres for each of the four methods and are summarized inFigures 4and5, respectively. Although SCS-CNN was the only method to have a nucleus with the “almost perfect agreement” classification, it had a wider distribution of classifications. Similar distributions were seen for FreeSurfer and T1-THOMAS, while HIPS-THOMAS was the only approach to have eight of 10 nuclei in the “substantial agreement” classification group. For the left hemisphere, HIPS-THOMAS had the highest Dice coefficient for 4 nuclei (VLp, VPL, LGN, and CM), FreeSurfer for 4 nuclei (AV, VA, MGN, and MD-Pf), and SCS-CNN for 2 nuclei (VLa, Pul). For the right hemisphere, HIPS-THOMAS had the highest Dice coefficient for 5 nuclei (AV, VA, VLp, VPL, and MD), FreeSurfer for 2 nuclei (VLa, CM), and SCS-CNN for 2 nuclei (MGN, Pul). Nuclei had smaller Average Hausdorff Distances along the diagonal (same nuclei) across segmentation approaches, except for left VLa for FreeSurfer, which bilaterally was closer to VLp, suggesting FreeSurfer is not able to distinguish well between VLa and VLp.

**Fig. 4.**
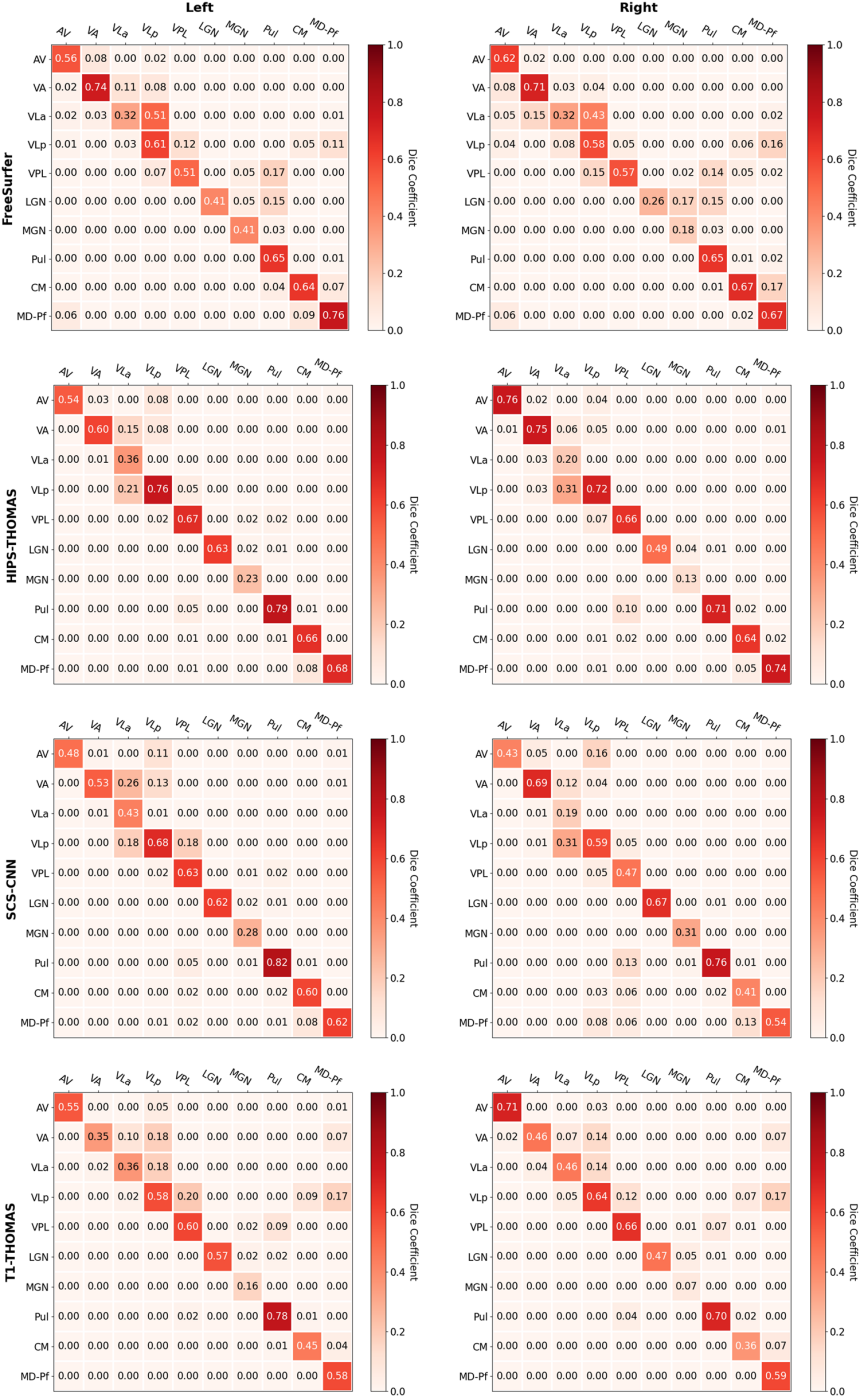
Group-level Dice overlap coefficients for nuclei segmented from Human Connectome Project data using FreeSurfer, HIPS-THOMAS, CNN-SCS, and T1-THOMAS approaches in MNI-space.

**Fig. 5.**
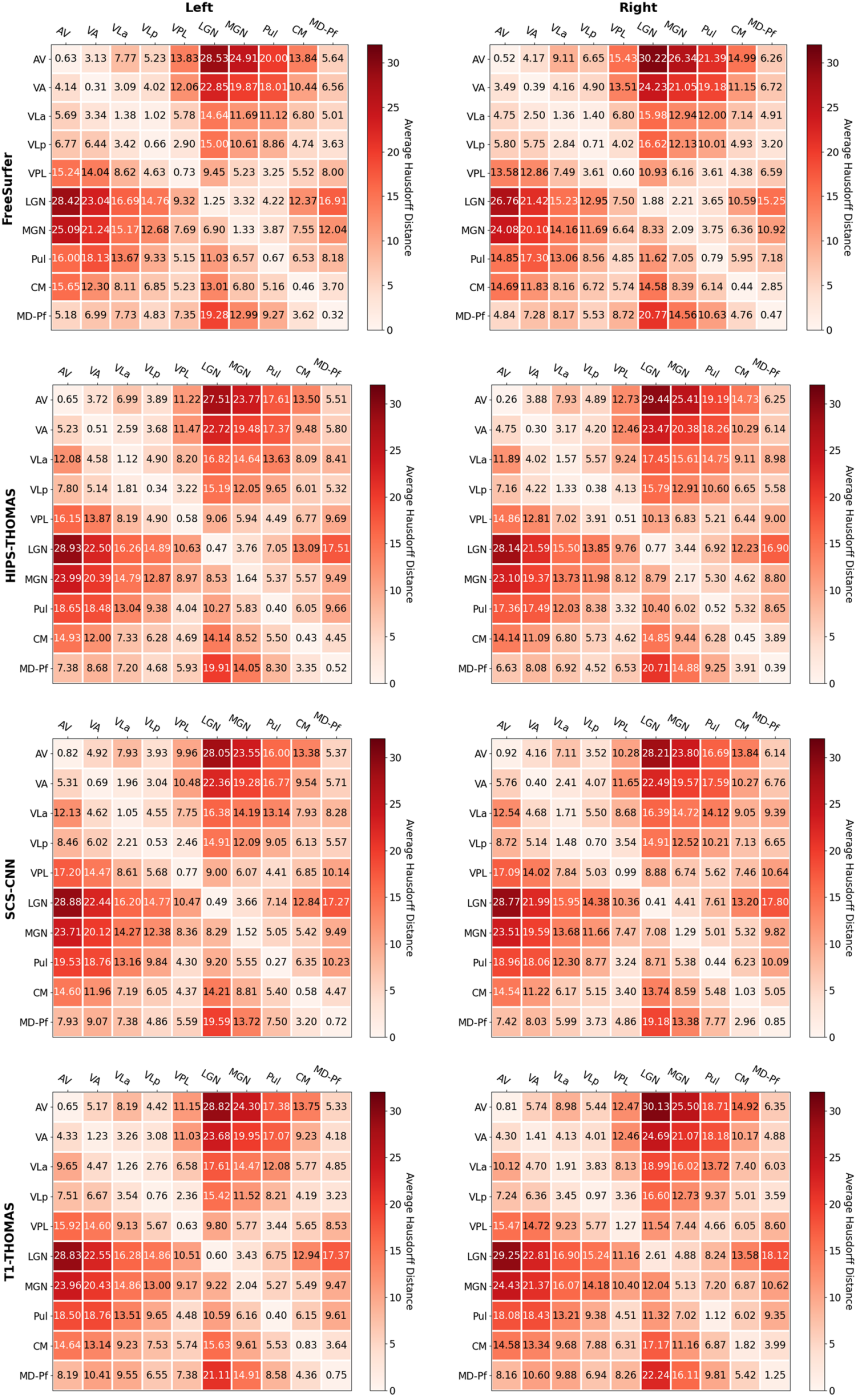
Group-level Average Hausdorff Distance for nuclei segmented from Human Connectome Project data using FreeSurfer, HIPS-THOMAS, CNN-SCS, and T1-THOMAS approaches in MNI-space.

#### 3.1.3 Interim summary: Human connectome project

The “best” method for each nucleus based on subject- and MNI-space Dice and AHD coefficients is graphically depicted inFigure 6(see “identifying nucleus-wise ‘best’ segmentation methods” for a detailed definition). The best performing methods were broadly similar for both subject- and MNI-space analyses, as reflected by the selection of a single nucleus in the right hemisphere, and all but three nuclei in the left hemisphere. In the left hemisphere, HIPS-THOMAS and FreeSurfer/SCS-CNN were joint best for CM and LGN respectively, while HIPS-THOMAS, FreeSurfer, and T1-THOMAS had comparable Dice coefficients for AV.

**Fig. 6.**
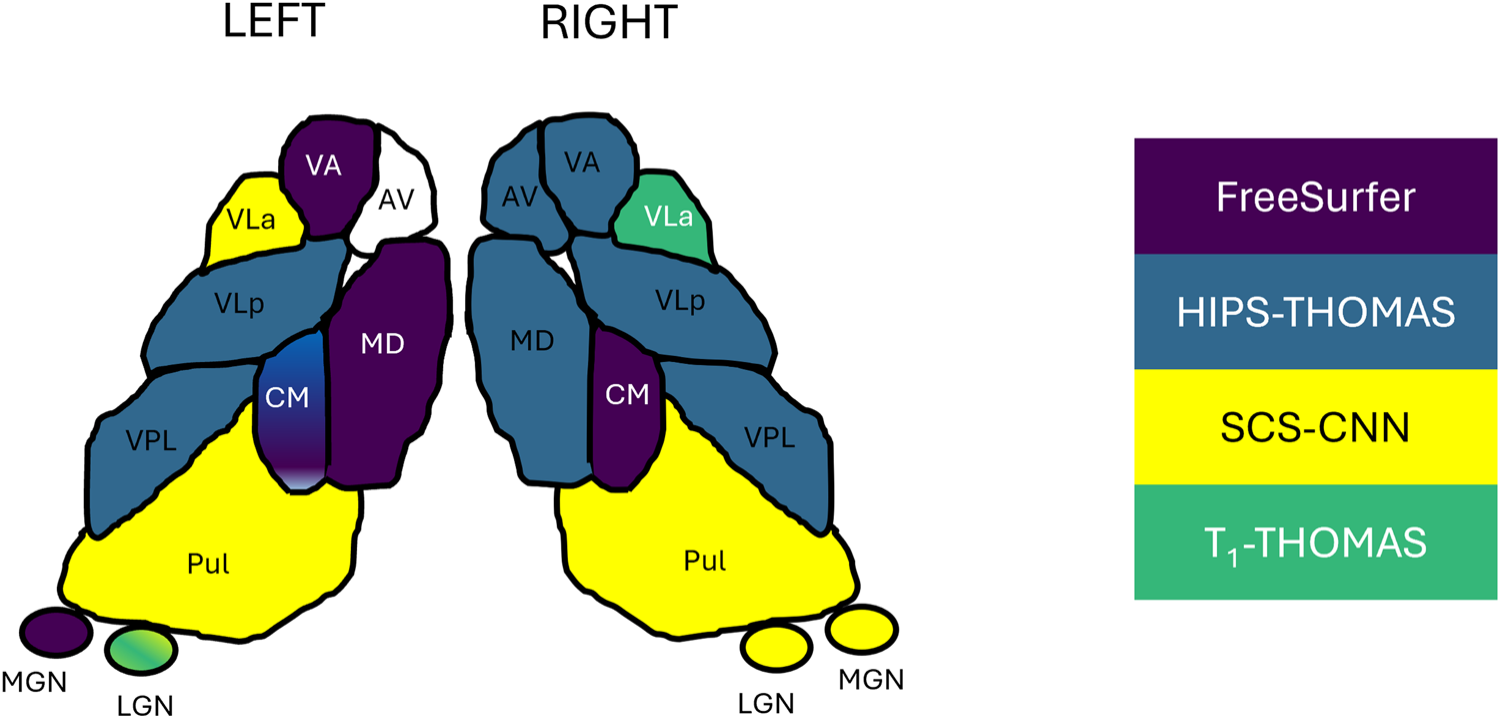
Best segmentation approach for each of the thalamic nuclei in each hemisphere, based on subject-space and group Dice and AHD coefficients.

### 3.2 Alzheimer’s disease neuroimaging initiative

**Fig. 7.**
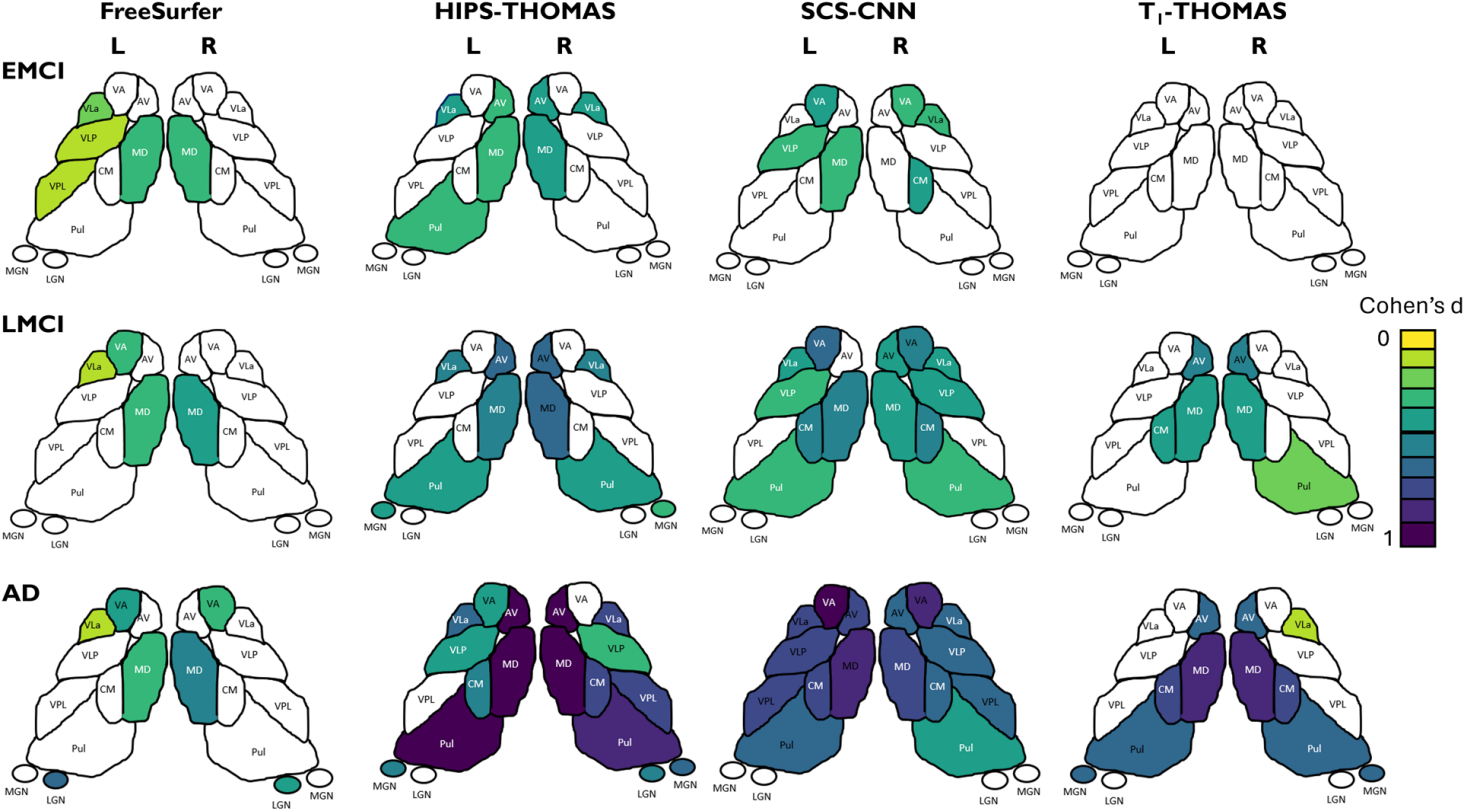
Thalamic nuclei atrophy as a function of AD stage (early mild cognitive impairment (EMCI), late mild cognitive impairment (LMCI), and Alzheimer’s disease (AD)) for the 4 segmentation methods. Nuclei with statistically significant different volumes between AD stage and healthy controls are colorized using effect size (Cohen’s d).

Figure 7shows thalamic nuclei atrophy colorized using Cohen’s d for the left and right hemispheres respectively, for HC-EMCI, HC-LMCI, and HC-AD comparisons. Only nuclei with statistically significant differences in the ANCOVA tests are coloured. The Cohen’s d provides a dimensionless metric for comparisons across methods. The progression of atrophy from EMCI to LMCI is captured nicely by SCS-CNN and HIPS-THOMAS, while FreeSurfer and T1-THOMAS do not exhibit a clear progression from EMCI to LMCI. Note that while SCS-CNN displays the progression, it was with reduced effect sizes.

The results of the ROC analyses are summarized inTable 2, and ROC curves are visualized inFigure 8. AUC values for discrimination of AD and HC for FreeSurfer, HIPS-THOMAS, SCS-CNN, and T1-THOMAS using all the individual thalamic nuclei volumes (adjusted for ICV/age) and whole thalamus volumes (for comparison) are shown. Classification accuracy using thalamic nuclei volumes was most accurate using HIPS-THOMAS while SCS-CNN was the most accurate when classifying using whole thalamus volumes, albeit with smaller AUCs for all three disease stages.

**Fig. 8.**
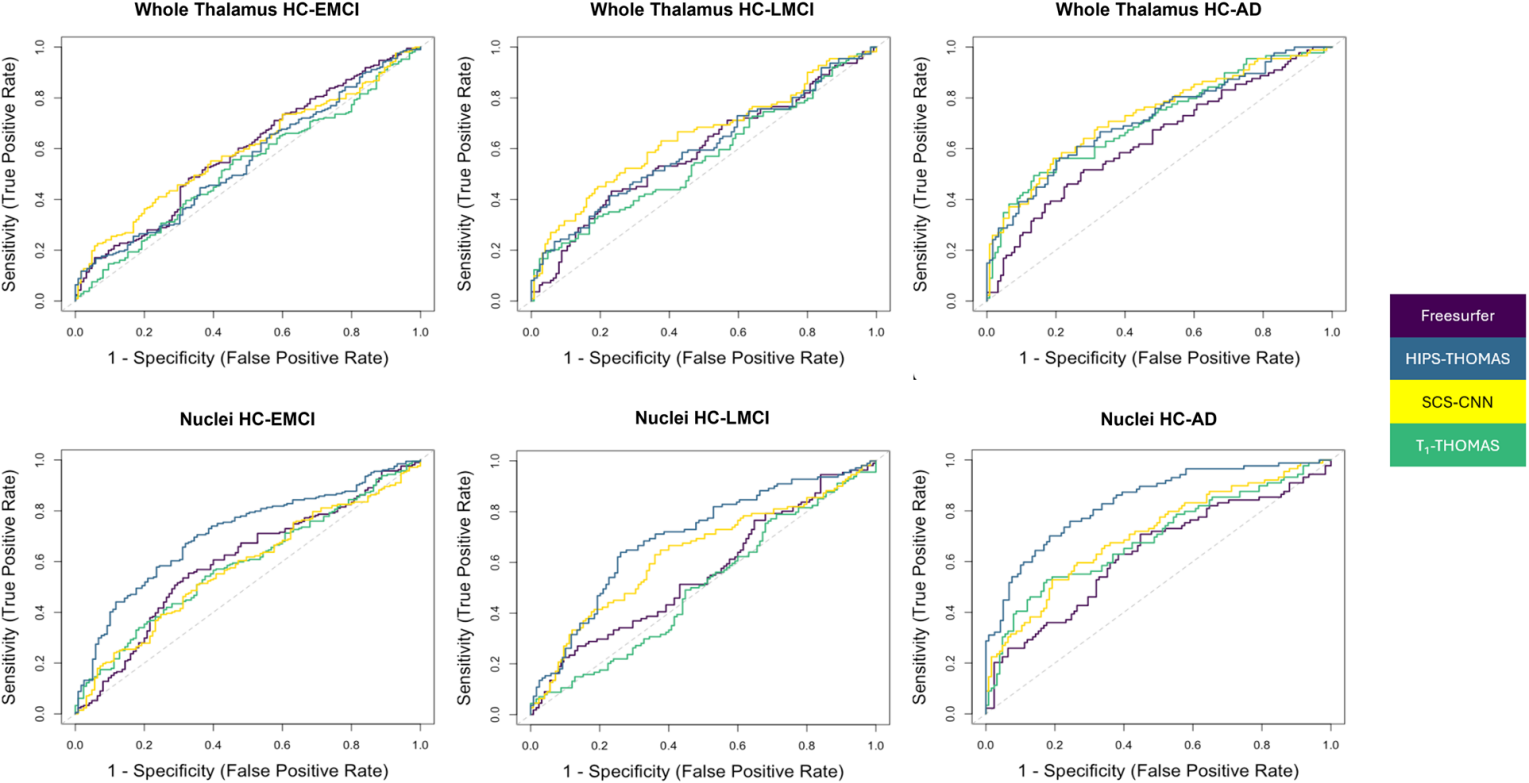
Receiver operating characteristic curves for the logistic regression used to quantify discriminability between those who are healthy and those with EMCI, LMCI, and AD using either whole thalamus or individual thalamic nuclei volumes. Discrimination using whole thalamus volume was best using SCS-CNN, while HIPS-THOMAS was best using individual nuclei volumes.

**Table 2.** Receiver operating characteristics analysis results.

	Freesurfer	HIPS-THOMAS	SCS-CNN	T1-THOMAS
HC-EMCI	Nuclei: 0.70Whole Thal: 0.60	Nuclei: 0.74*Whole Thal: 0.58	Nuclei: 0.69Whole Thal: 0.62†	Nuclei: 0.73Whole Thal: 0.61
HC-LMCI	Nuclei: 0.69Whole Thal: 0.62	Nuclei: 0.82*Whole Thal: 0.63	Nuclei: 0.76Whole Thal: 0.66†	Nuclei: 0.73Whole Thal: 0.65
HC-AD	Nuclei: 0.77Whole Thal: 0.66	Nuclei: 0.93*Whole Thal: 0.74	Nuclei: 0.85Whole Thal: 0.76†	Nuclei: 0.81Whole Thal: 0.72

AUC values for discriminating healthy controls from early MCI, late MCI, and AD using either individual nuclei or whole thalamus volumes are presented. The highest AUC value for each comparison is denoted for nuclei (*) and whole thalamus (†) segmentation.

## 4 Discussion

To date, several thalamic nuclei segmentation methods based on diffusion MRI, resting-state fMRI, and, more recently, structural MRI have been reported, but comparisons across different segmentation methods are almost non-existent. WhileIglehart et al. (2020)qualitatively compared a method from each of those 3 classes (i.e., diffusion, functional, and structural MRI) on a small cohort of 20 healthy subjects, this is the first work—to the best of our knowledge—that quantitatively compares state-of-the-art structural imaging-based thalamic nuclei segmentation methods, using two large cohorts to benchmark segmentation approaches using Dice and AHD coefficients, as well as AUC scores in a clinical context. For the HCP cohort, using both Dice and AHD as quantitative metrics, HIPS-THOMAS displayed the best performance (4/10 nuclei on left, 5/10 nuclei on right). These results were consistent across subject-space and MNI-space analyses. For the ADNI cohort, HIPS-THOMAS achieved the best AUC scores for discrimination between controls and all three AD disease stages—early MCI, late MCI, and AD—using individual thalamic nuclei volumes. While SCS-CNN achieved the best AUC scores using whole thalamic volumes for discrimination, these AUC values were lower than those achieved using thalamic nuclei, a result also observed for controls-AD discrimination byIglesias et al. (2018)using the FreeSurfer Bayesian parcellation.

One of the main issues hampering accurate segmentation of thalamic nuclei from standard T1w MRI data is the lack of intrathalamic contrast as well as the poor delineation of whole thalamic boundaries. Novel sources of contrast such as white-matter nulled contrast provided by FGATIR (Sudhyadhom et al., 2009) or WMn-MPRAGE (Tourdias et al., 2014) significantly improve intra-thalamic contrast. They also provide a good depiction of whole thalamus boundaries, especially the ventral boundaries which are adjacent to white-matter tracts. WMn contrast optimized at 7T was exploited by the original THOMAS method (Tourdias et al., 2014). The idea of synthesizing WMn-MPRAGE contrast in the absence of acquired WMn-MPRAGE data was first proposed byDatta et al. (2021), who showed that WMn-MPRAGE data synthesized from the T1 maps derived from the MP2RAGE acquisition improved Dice compared to direct segmentation of the MP2RAGE ratio image. Since MP2RAGE is still not commonly used at 3T and not available in public databases like HCP and ADNI, methods which directly synthesize WMn-MPRAGE-like images from T1w MRI like the SCS-CNN and HIPS-THOMAS used in this work were proposed. In our analyses, both SCS-CNN and HIPS-THOMAS showed significantly improved Dice compared to FreeSurfer or T1-THOMAS for the ventral nuclei, suggesting the utility of improved delineation of the ventral thalamic borders enabled by the WMn contrast. The geniculate nuclei also showed significant improvements. FreeSurfer showed better Dice performance in the medial thalamus, specifically in the left mediodorsal nucleus and bilateral centromedian nucleus. One plausible reason for this difference is that because the medial thalamus shares a boundary with the third ventricle, the contrast between grey matter and CSF would be greater for traditional CSF-nulled contrast (i.e., standard MPRAGE) than WMn-MPRAGE where CSF is grey and not black. In turn, this contrast may make delineating nuclei more efficacious using standard MPRAGE as for FreeSurfer, than methods using (synthesized) WMn-MPRAGE as is the case for the SCS-CNN and HIPS-THOMAS methods. Both the WMn-synthesis-based methods (HIPS-THOMAS and SCS-CNN) achieved larger effect sizes and captured the progress of atrophy from EMCI to AD better than T1-THOMAS or Freesurfer. One major advantage HIPS-THOMAS offers over SCS-CNN (besides the larger AUC for nuclei-based discrimination) is its robustness. CNNs are very sensitive to training data and that was the case for SCS-CNN, which performed sub-optimally on Philips 3T and Siemens 7T data relative to the Siemens and GE 3 T data it was trained on (Umapathy et al., 2022). In contrast, the simpler polynomial-based method performed more robustly on all data inputs, something which is critical when analyzing large public databases which often contain a mixture of field strengths and scanner types.

Iglesias et al. (2018)compared thalamic nuclei volumes from their Freesurfer probabilistic atlas-based parcellation against volumes segmented using the Krauth-Morel atlas as a sort of first-level validation. Six representative nuclei were used to make qualitative comparisons of volume distributions on 66 subjects, and a qualitative visual overlap with Krauth-Morel atlas was described. A more quantitative comparison using Dice and AHD was reported byWilliams et al. (2022)on 100 HCP subjects. In this work, we followed the same approach as both of these works with a further harmonization of nuclei across Freesurfer and THOMAS to enable direct comparisons of these methods against the Krauth-Morel atlas. A replication of the Dice and AHD analyses comparing THOMAS variants against FreeSurfer (instead of Krauth-Morel) is presented inSupplementary Figures 4–7. Both sets of analyses have to be interpreted with caution. The Freesurfer nuclei labeling is based on theJones (2012)nomenclature, which, while largely similar to the Morel nomenclature, has slight differences (anterior medial and dorsal merged with ventral for example). Even though Freesurfer is one of the most commonly used methods for thalamic nuclei segmentation of T1 data, as pointed above, it has not been validated rigorously against manual segmentation ground truth. A limited analysis against manual segmentation ground truth showed poor results in several nuclei such as LGN and MGN compared to THOMAS (Su et al., 2019) and inconsistencies when compared to Krauth-Morel (Williams et al., 2022). The relative performance of three THOMAS variants was similar whether using Krauth-Morel or Freesurfer.

Our work had several limitations. Firstly, although individual manual segmentation should be considered as the “gold” standard for creating a reference for benchmarking automated thalamic segmentation approaches, due to the impracticalities of manually labeling 640 subjects, we instead used the Krauth-Morel atlas as a surrogate “silver” standard. Secondly, due to the differences in nomenclature as well as the nuclei segmented by FreeSurfer and THOMAS plus its variants, we created a set of 10 common nuclei by merging some nuclear subdivisions such as within pulvinar and mediodorsal nuclei, to enable equivalent comparisons across methods. Thirdly, this work evaluated four of the main anatomical-based segmentation methods, but did not consider all anatomical methods, or functional- or diffusion-based approaches. However, functional- and diffusion-based approaches produce segmentations that are based on statistical and diffusivity properties, respectively, and therefore are fundamentally different approaches to generating segmentations. Despite these aforementioned limitations, many published segmentation approaches make qualitative and semi-quantitative comparisons with respect to the Morel atlas. Therefore, unifying the principle anatomical segmentation methods under a single nomenclature and comparison with the Krauth-Morel atlas provides greater insight into which methods are the most similar to the Morel atlas as a standard reference space.

Future work should aim to investigate how to best combine and make use of the information generated by both SCS-CNN and HIPS-THOMAS approaches, since they have complementary advantages with respect of accuracy of segmentation for different nuclei, and overall robustness. This is of particular importance for enquiries into the functional role of thalamic nuclei in both health and disease (Shine et al., 2023). For instance, existing large datasets, such as the Human Connectome Project (Van Essen et al., 2013), Adolescent Brain Cognitive Development study (Casey et al., 2018), and the UK Biobank (Sudlow et al., 2015), provide opportunities for statistically well-powered work with high-quality data from many subjects. However, large neuroimaging datasets often only include T1 structural images. Therefore, it is important to continue developing our understanding of using the T1 signal for thalamic segmentation to enhance the utility of datasets without optimized acquisitions such as WMn for thalamic function research. Future work would also benefit from developing tools that include information from other imaging modalities, as is the case for the recent improvement to the FreeSurfer based segmentation approach, which combines diffusion data with T1 images (Tregidgo et al., 2023). For example, other imaging contrasts which may benefit thalamic segmentation include magnetization transfer, which improves thalamic contrast (Gringel et al., 2009), and proton density, which enhances contrast between the lateral geniculate and surrounding white matter (Fujita et al., 2001). Lastly, though we identify volumetric changes in thalamic nuclei volume that are associated with Alzheimer’s disease progression, discussing its relevance to the aetiology of Alzheimer’s pathophysiology is beyond the scope of this work. However, recent work has demonstrated that individuals with prodromal autosomal dominant Alzheimer’s disease have differential associations between thalamic nuclei volume and amyloid/tau pathology when compared with healthy controls (Pardilla-Delgado et al., 2021). These differences exist in spite of a lack of aging-related pathology, and demonstrate the importance of understanding how changes to thalamic nuclei volume and function contribute towards Alzheimer’s disease and its progression (Forno et al., 2023).

## Supplementary Material

Supplementary Material

## Data Availability

Data were provided by the Human Connectome Project, WU-Minn Consortium (Principal Investigators: David Van Essen and Kamil Ugurbil; 1U54MH091657) funded by the 16 NIH Institutes and Centers that support the NIH Blueprint for Neuroscience Research; and by the McDonnell Center for Systems Neuroscience at Washington University. Data were also obtained from the Alzheimer’s Disease Neuroimaging Initiative (ADNI) database (https://adni.loni.usc.edu). The ADNI was launched in 2003 as a public–private partnership, led by Principal Investigator Michael W. Weiner, MD. FreeSurfer segmentation was completed using code previously reported inWilliams et al. (2022), and is available online at the University of Reading Research Data Archive (https://doi.org/10.17864/1947.000339). Details for running T1-THOMAS and HIPS-THOMAS using Docker are available on Github (T1-THOMAShttps://github.com/thalamicseg/thomas_new, HIPS-THOMAShttps://github.com/thalamicseg/hipsthomasdocker); the Docker images are available on Docker Hub (https://hub.docker.com/u/anagrammarian). The Docker image for running SCS-CNN is available on Github (https://github.com/lunastra26/thalamic-nuclei-segmentation).
