# Supplementary material for: Thalamic nuclei segmentation from T1-weighted MRI: Unifying and benchmarking state-of-the-art methods
Source: Imaging Neurosci (Camb). 2024 May 8;2:imag-2-00166. doi: 10.1162/imag_a_00166 (PMC11873765; doi:10.1162/imag_a_00166)
Supplement: Supplementary Material [file imag_a_00166-supp.pdf]

Supplementary methods

THOMAS pipeline and its variants: The original THOMAS method that was developed and optimized for WMn-MPRAGE uses a set of 20 WMn-MPRAGE datasets (p_1-p_{20}) as priors which have been manually segmented using the Moral atlas as guide. The 20 priors are mutually registered and averaged to create a WMn template. The input image is first cropped and registered to a cropped WMn template image using ANTs nonlinear registration (R). The precomputed prior-to-template space warps (W_{piT}) are combined with R^{-1} to warp the 20 prior labels to input space. These labels are then combined using a joint-fusion algorithm to generate a single parcellation in subject space. The WMn-MPRAGE sequence is neither part of standard clinical imaging protocols nor part of extant databases such as ADNI and OASIS. To adapt THOMAS for T1w data, one approach was to replace the cross-correlation (CC) metric with a mutual information (MI) metric in the ANTs nonlinear registration step of THOMAS and replace the joint fusion (JF) with majority voting (MV) in the label fusion step of THOMAS. We refer to this variant as T1-THOMAS. To leverage the improved intrathalamic contrast of WMn-MPRAGE, a polynomial synthesis method (box labelled HIPS) was used to first synthesize WMn-MPRAGE-like images from T1w images before applying the THOMAS algorithm. Note that the WMn-like input enables the use of the more accurate CC metric for nonlinear registration as well as the more sophisticated JF algorithm compared to MV for label fusion. We call this method HIPS-THOMAS. The original THOMAS method and the T1-THOMAS and HIPS-THOMAS variants are shown in Supplemental Figure 1 below, using green, red, and cyan colours to differentiate the three methods.

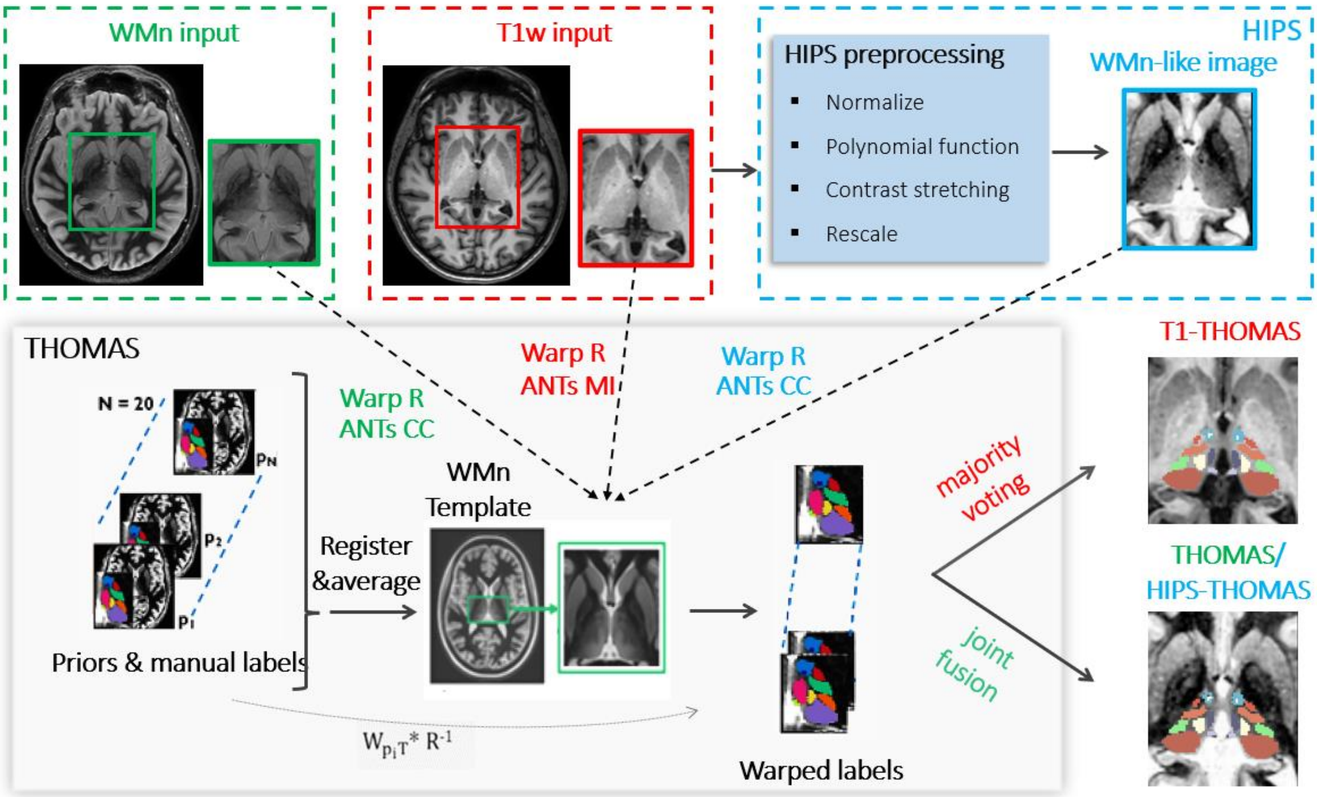

Supplementary Figure 1. Schematic of THOMAS and the two variants- T1-THOMAS and HIPS-THOMAS. T1-THOMAS (grey text) uses a mutual information metric for nonlinear registration of input to template and a majority voting algorithm to combine the labels. HIPS-THOMAS (cyan text) uses a cross-correlation metric for more accurate nonlinear registration of input to template and a joint fusion algorithm for label fusion.

Supplementary results

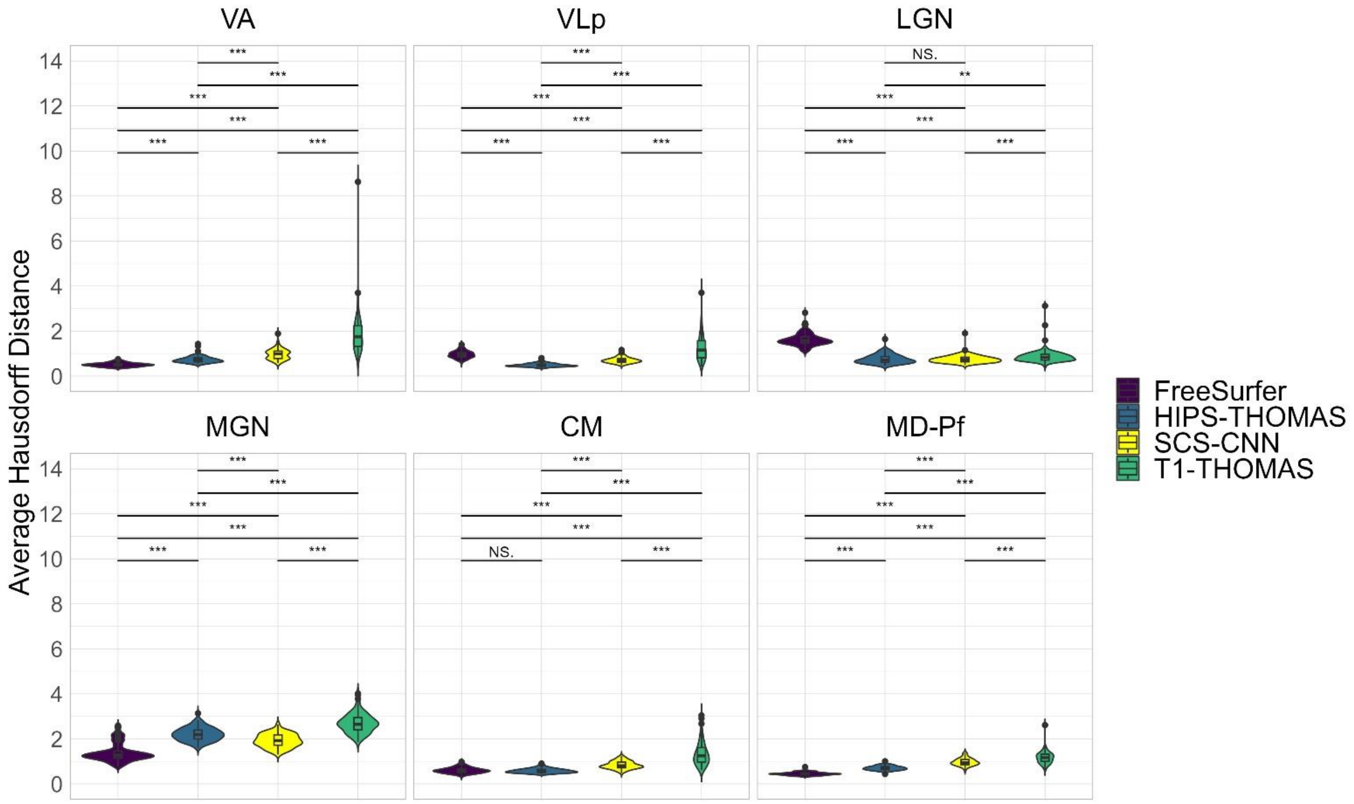

Supplementary Figure 2. Violin plots of left hemisphere nuclei with significantly different Average Hausdorff Distances for nuclei segmented from Human Connectome Project data using FreeSurfer, HIPS-THOMAS, CNN-SCS, and T1-THOMAS approaches. Posthoc t-test results (Bonferroni corrected) are presented to show pairwise difference between segmentation approaches for each nucleus (**p<0.01, ***p<0.001).

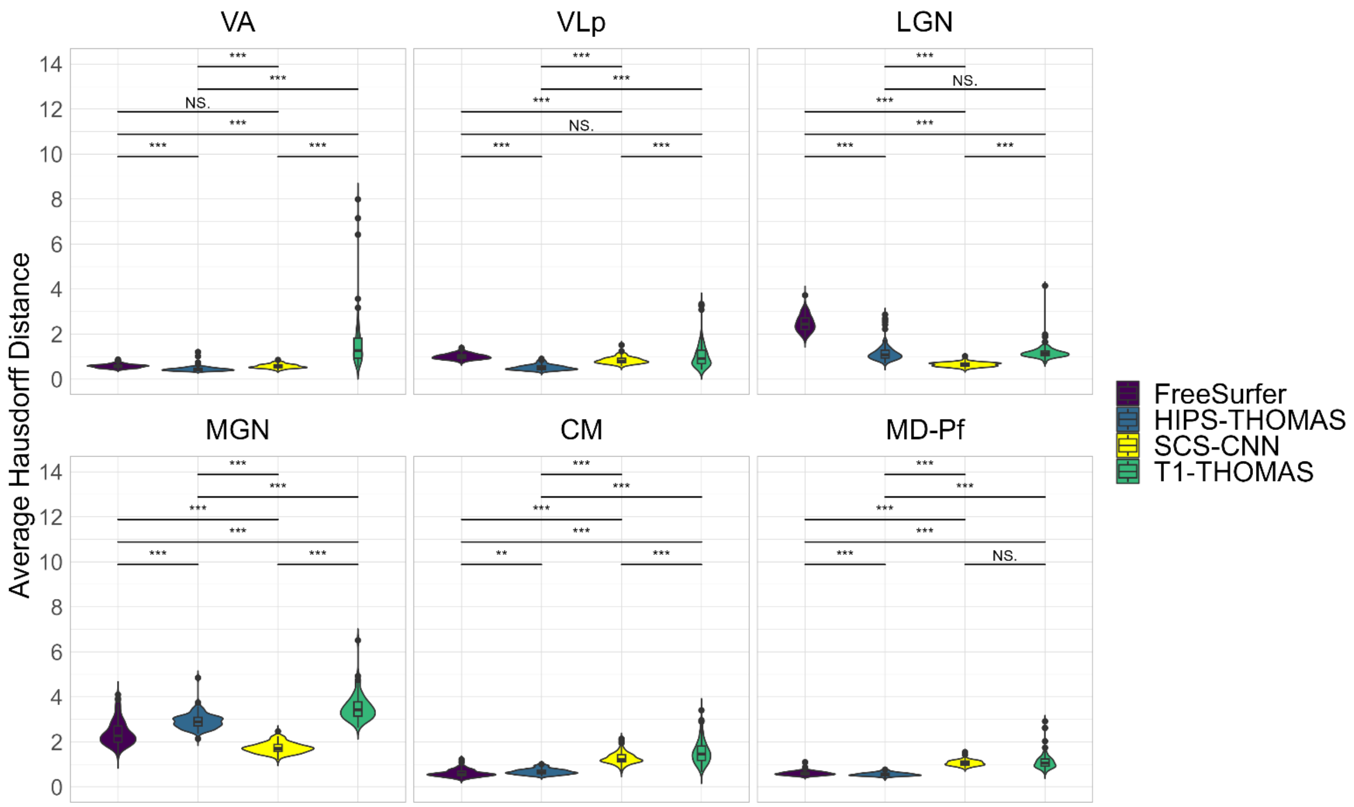

Supplementary Figure 3. Violin plots of right hemisphere nuclei with significantly different Average Hausdorff Distances for nuclei segmented from Human Connectome Project data using FreeSurfer, HIPS-THOMAS, CNN-SCS, and T1-THOMAS approaches. Posthoc t-test results (Bonferroni corrected) are presented to show pairwise difference between segmentation approaches for each nucleus (**p<0.01, ***p<0.001).

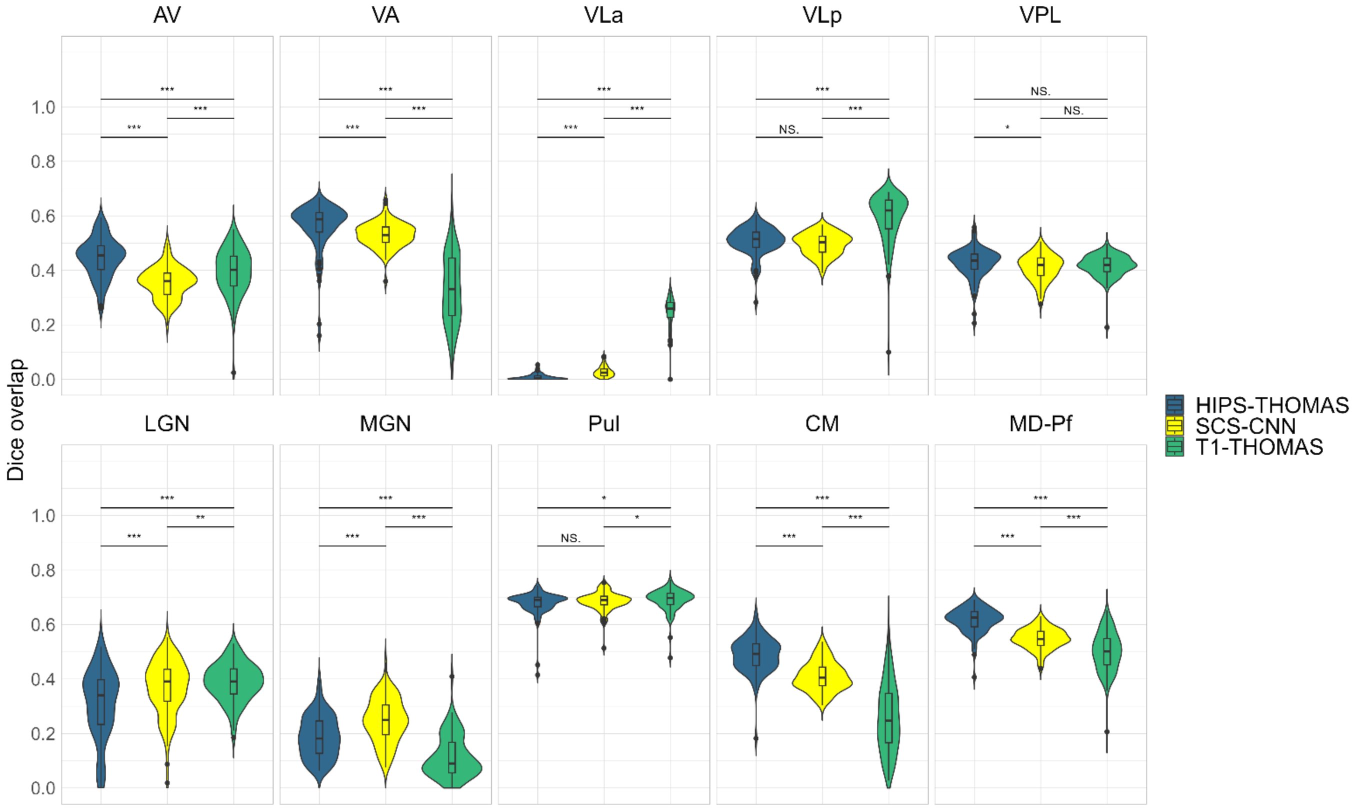

Supplementary Figure 4. Violin plots of left hemisphere Dice overlap using Freesurfer as a reference space for THOMAS-variants with Human Connectome Project data. Posthoc t-test results (Bonferroni corrected) are presented to show pairwise difference between segmentation approaches for each nucleus (*p<0.05, **p<0.01, ***p<0.001).

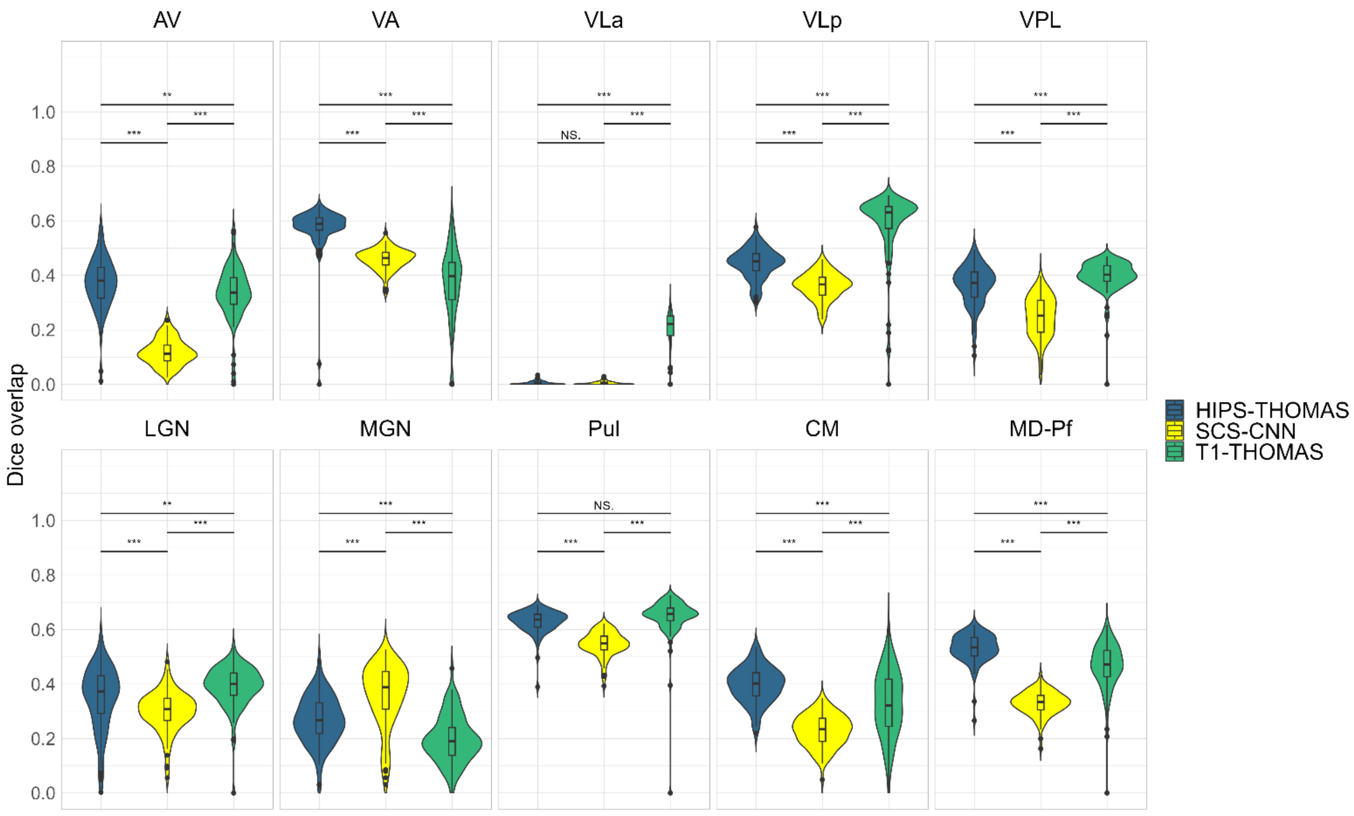

Supplementary Figure 5. Violin plots of right hemisphere Dice overlap using Freesurfer as a reference space for THOMAS-variants with Human Connectome Project data. Posthoc t-test results (Bonferroni corrected) are presented to show pairwise difference between segmentation approaches for each nucleus (*p<0.05, **p<0.01, ***p<0.001).

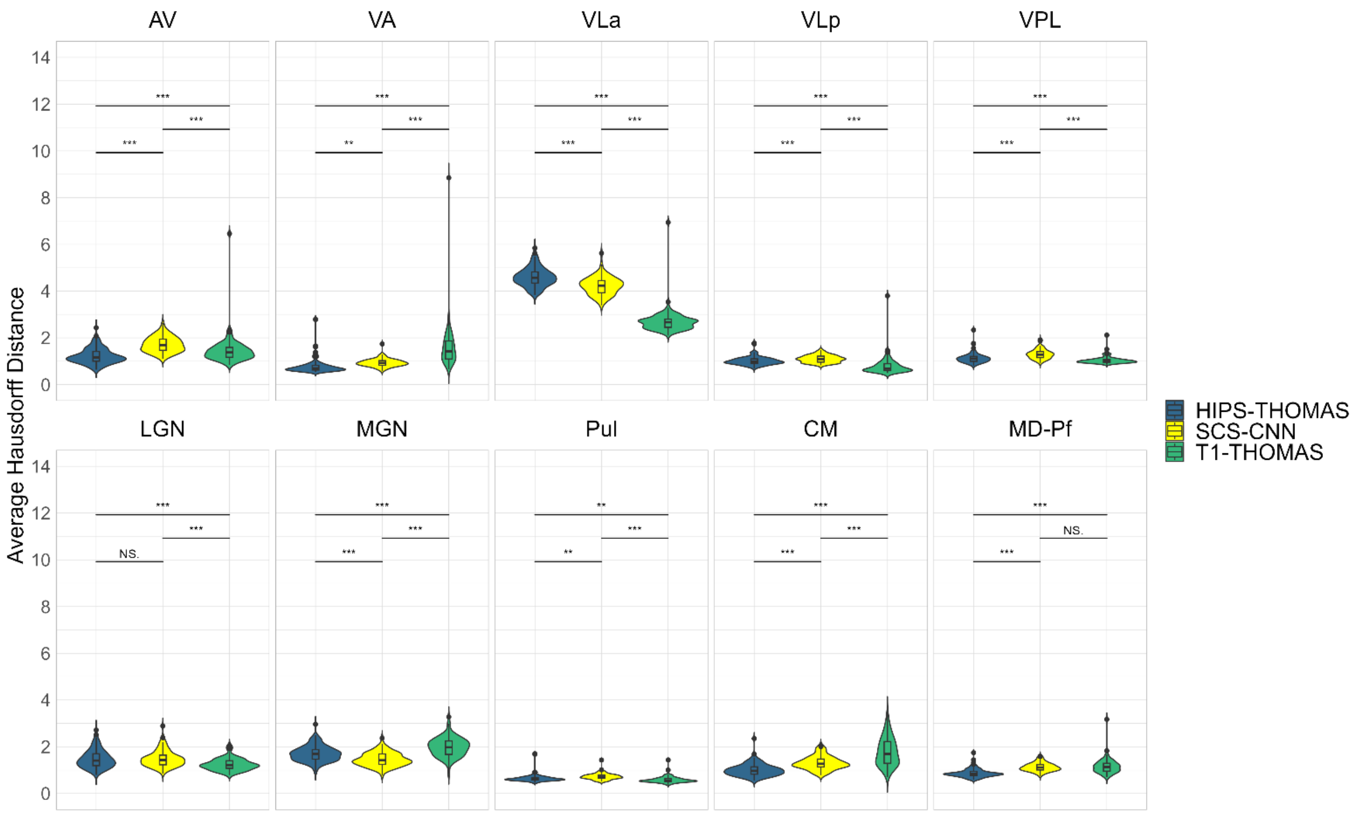

Supplementary Figure 6. Violin plots of left hemisphere Average Hausdorff Distance using Freesurfer as a reference space for THOMAS-variants with Human Connectome Project data. Posthoc t-test results (Bonferroni corrected) are presented to show pairwise difference between segmentation approaches for each nucleus (*p<0.05, **p<0.01, ***p<0.001).

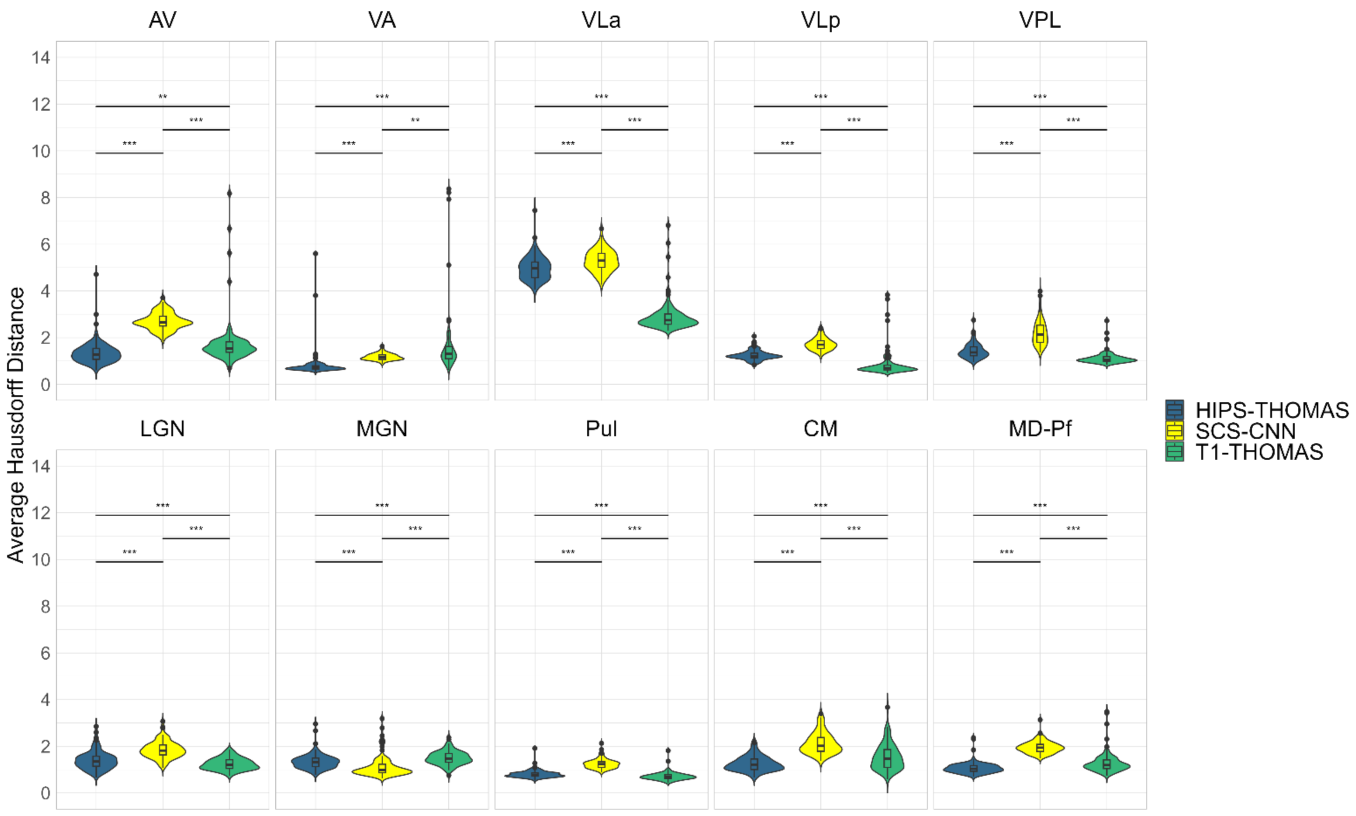

Supplementary Figure 7. Violin plots of right hemisphere Average Hausdorff Distance using Freesurfer as a reference space for THOMAS-variants with Human Connectome Project data. Posthoc t-test results (Bonferroni corrected) are presented to show pairwise difference between segmentation approaches for each nucleus (*p<0.05, **p<0.01, ***p<0.001).

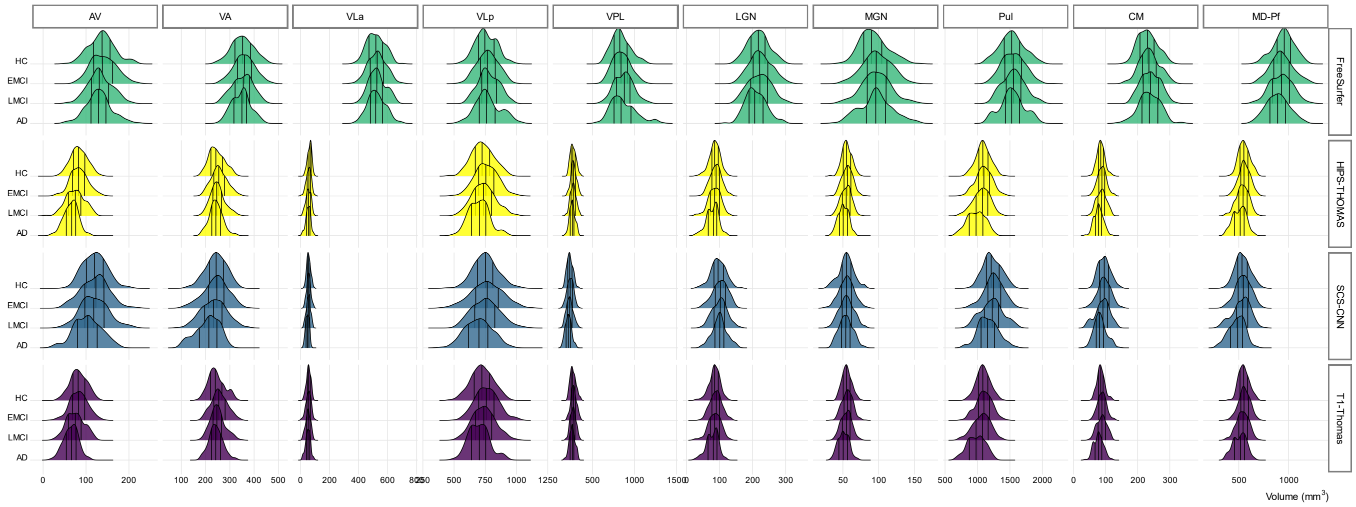

Supplementary Figure 8. Density plots for volumes of segmented thalamic nuclei for data from healthy controls (HC), early minor cognitive impairment (EMCI), late minor cognitive impairment (LMCI), and Alzheimer's disease (AD) using the 4 segmentation methods. Vertical lines for each density plot represent quantiles.

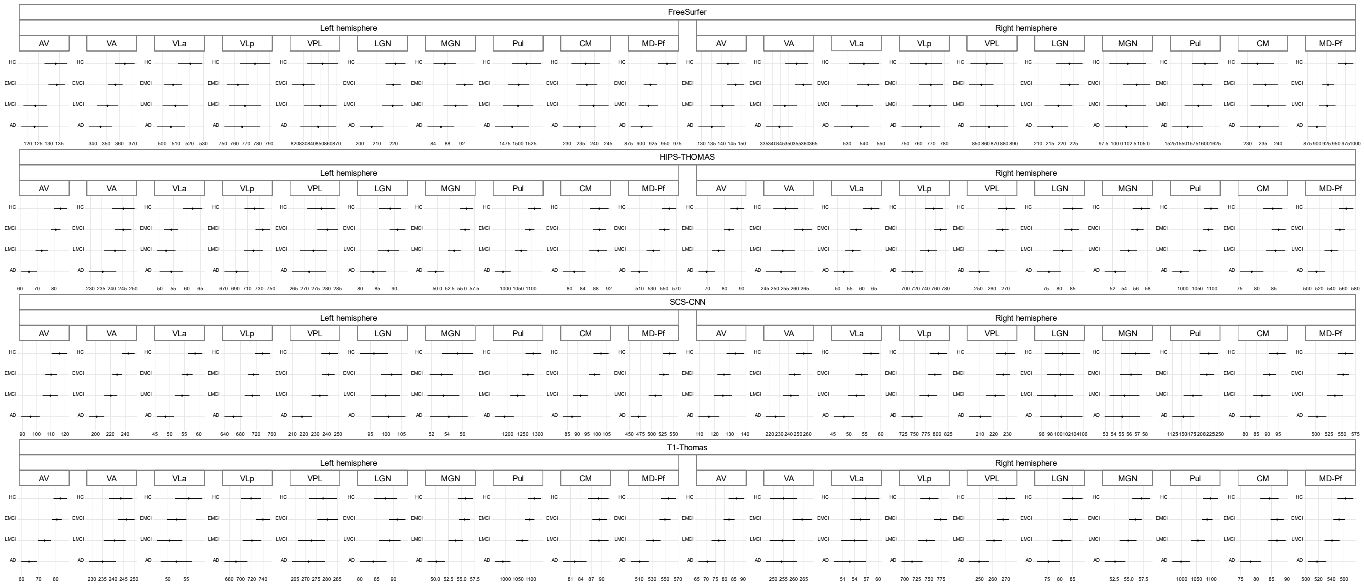

Supplementary Figure 9. Estimated marginal means for volumes of segmented thalamic nuclei for data from healthy controls (HC), early minor cognitive impairment (EMCI), late minor cognitive impairment (LMCI), and Alzheimer’s disease (AD) using the 4 segmentation methods. Estimated marginal means were calculated from ANCOVAs used to compare nuclei volumes for each segmentation method

Supplementary table 1. Two-way ANOVA results for HCP dataset analysis in subject space for each nucleus. Significant main effects of segmentation approach (dataset) and hemisphere (side), and interactions were found for all nuclei except for VPL, which did not show a main effect of side.

Effect	DFn	DFd	F	p	p<.05	ges	segmentation
Dataset	2.56	251.05	138.753	3.06E-48	*	0.333	AV
side	1	98	156.403	5.10E-22	*	0.196	AV
Dataset:side	1.9	185.8	110.71	2.50E-31	*	0.199	AV
Dataset	1.35	132.2	596.277	1.24E-57	*	0.719	VA
side	1	98	286.446	7.60E-31	*	0.213	VA
Dataset:side	1.83	179.69	100.781	1.75E-28	*	0.126	VA
Dataset	2.11	206.35	85.01	1.30E-28	*	0.31	VLa
side	1	98	79.947	2.40E-14	*	0.059	VLa
Dataset:side	1.97	192.81	166.994	2.08E-42	*	0.251	VLa
Dataset	1.56	153.36	184.886	1.79E-36	*	0.498	VLp
side	1	98	18.759	3.59E-05	*	0.016	VLp
Dataset:side	1.47	143.99	55.408	3.26E-15	*	0.08	VLp
Dataset	1.72	168.96	75.557	5.91E-22	*	0.275	VPL
side	1	98	0.692	0.407		0.000709	VPL
Dataset:side	1.79	175.34	56.634	2.78E-18	*	0.1	VPL
Dataset	2.74	268.54	407.59	1.34E-95	*	0.593	LGN
side	1	98	357.482	1.83E-34	*	0.267	LGN
Dataset:side	2.57	252.24	92.152	1.79E-36	*	0.202	LGN
Dataset	1.25	122.32	188.742	5.26E-30	*	0.515	MGN
side	1	98	698.738	2.18E-46	*	0.35	MGN
Dataset:side	1.47	143.66	126.936	3.58E-27	*	0.2	MGN
Dataset	2.07	202.89	490.928	1.16E-79	*	0.531	Pul

side	1	98	268.92	7.54E-30	*	0.23	Pul
Dataset:side	1.56	152.6	35.716	2.16E-11	*	0.048	Pul
Dataset	1.65	161.51	523.075	7.27E-66	*	0.74	CM
side	1	98	277.192	2.52E-30	*	0.127	CM
Dataset:side	2.09	205.2	80.828	1.95E-27	*	0.101	CM
Dataset	1.62	159.22	641.411	5.30E-71	*	0.758	MD-Pf
side	1	98	37.576	1.85E-08	*	0.036	MD-Pf
Dataset:side	1.58	155.05	147.22	5.01E-32	*	0.202	MD-Pf

76
